# Determinants of Health-Related Quality of Life in Women with Turner Syndrome: The Role of Comorbidities, Hormonal Therapy and Depressive Symptoms

**DOI:** 10.3390/jcm15031088

**Published:** 2026-01-30

**Authors:** Mariola Krzyścin, Ewelina Soszka-Przepiera, Katarzyna Zając, Agnieszka Brodowska, Adam Przepiera, Dominika Pietrzyk, Žana Bumbulienė, Elżbieta Sowińska-Przepiera

**Affiliations:** 1 Pediatric, Adolescent Gynecology Clinic, Department of Gynecology, Endocrinology and Gynecological Oncology, Pomeranian Medical University in Szczecin, Unii Lubelskiej 1, 71-252 Szczecin, Poland; 2 II Department of Ophthalmology, Pomeranian Medical University, Al. Powstańców Wlkp. 72, 70-111 Szczecin, Poland; 3 Department of Gynecology, Endocrinology and Gynecological Oncology, Pomeranian Medical University in Szczecin, Unii Lubelskiej 1, 71-252 Szczecin, Poland; 4 Department of Urology and Urologic Oncology, Pomeranian Medical University in Szczecin, Powstańców Wielkopolskich 72, 70-111 Szczecin, Poland; 5 Clinic of Obstetrics and Gynecology, Institute of Clinical Medicine, Faculty of Medicine, Vilnius University, LT-08661 Vilnius, Lithuania; 6 Individual Laboratory for Endocrine Diagnostics, Pomeranian Medical University in Szczecin, Unii Lubelskiej 1, 71-252 Szczecin, Poland

**Keywords:** Turner syndrome, quality of life, psychosocial functioning

## Abstract

**Background**: Turner syndrome (TS) is a chromosomal disorder associated with considerable phenotypic variability and lifelong multisystem comorbidities. Beyond somatic manifestations, TS may substantially affect physical, psychological, and social functioning, highlighting the need for comprehensive assessment of quality of life in affected women. **Methods**: This observational comparative study included 30 adult women with genetically confirmed Turner syndrome and 43 age-matched healthy controls. Quality of life was assessed using the SF-36 questionnaire, with clinical, anthropometric, and psychosocial variables analyzed as potential predictors using correlation and multivariable regression analyses. **Results**: Women with Turner syndrome were significantly shorter than controls and more frequently affected by hypothyroidism, cardiac defects, and hearing impairment. They scored lower on SF-36 domains of general health, vitality, social functioning, and mental health, while exhibiting higher BDI-II depressive symptoms. Quality of life correlated negatively with comorbidity burden and depressive symptoms, positively with final height, and was lower in patients with hearing impairment, highlighting the multifactorial determinants of well-being in TS. **Conclusions**: Health-related quality of life in women with Turner syndrome is shaped by a complex interplay of somatic burden, psychological well-being, and social functioning. Depressive symptoms, comorbidities, stature, and hearing impairment significantly influence outcomes, emphasizing the need for holistic, multidisciplinary care that extends beyond medical management.

## 1. Introduction

Turner syndrome (TS) is a chromosomal disorder resulting from complete or partial monosomy of the X chromosome, most commonly manifesting as the 45, X karyotype, although mosaicism and structural abnormalities of the X chromosome are also observed [1]. The phenotype of TS is highly heterogeneous and includes characteristic dysmorphic features, short stature, and gonadal dysgenesis leading to infertility, alongside an increased risk of cardiovascular, endocrine, renal, otolaryngological, and ophthalmological comorbidities. Despite its well-recognized clinical picture, considerable variability in presentation persists, suggesting the involvement of additional genetic and epigenetic mechanisms beyond simple chromosomal imbalance.

The clinical features of Turner syndrome were first noted as early as 1761, when Morgagni [2] described a woman with short stature, primary amenorrhea, and a webbed neck—now recognized as hallmark characteristics. The modern clinical understanding developed gradually in the early 20th century: Shereshevski (1925) and Ullrich (1930) [3,4] reported cases of short stature, sexual infantilism, and dysmorphic features, and Turner (1938) [5] synthesized these findings into a distinct clinical syndrome characterized by absent puberty and gonadal dysgenesis. The eponym “Turner syndrome” was introduced in 1941 by Sharpey-Schafer [6]. Its chromosomal basis—monosomy X—was established in the 1950s, following cytogenetic discoveries by Ford, Polani, and others, marking TS as the first human disorder linked to a specific chromosomal abnormality.

TS occurs in approximately 1 in 2500 live female births [7], although prevalence is likely underestimated due to unrecognized mild cases [8,9,10,11]. The classic 45, X karyotype appears in roughly 3% of conceptions, but 99% of affected fetuses miscarry, accounting for nearly 10% of all pregnancy losses. Population statistics vary geographically, with studies from Europe, the US, and Poland providing prevalence estimates between 17 and 35 per 100,000 girls and women [12,13]. Modern diagnostic practice increasingly involves prenatal detection via non-invasive prenatal testing (NIPT), although positive screenings require cytogenetic confirmation through invasive testing such as amniocentesis or chorionic villus sampling. Prenatal ultrasound findings suggestive of TS include increased nuchal translucency, cystic hygroma, generalized oedema, coarctation of the aorta, bicuspid aortic valve, renal abnormalities, and growth restriction [14,15]. High prenatal detection rates correlate with elevated termination rates—up to 76–84% in several European countries [16,17].

Life expectancy in TS is reduced by 13–15 years, with cardiovascular disease accounting for approximately half of the excess mortality, followed by endocrine, neurological, and hepatic complications. Although overall cancer risk does not appear increased, melanomas and central nervous system tumors occur more frequently in this population [18].

Clinically, TS should be suspected in newborns or girls presenting with lymphedema, short stature, delayed puberty, or characteristic dysmorphic traits such as low-set ears, epicanthal folds, a webbed neck, shield chest, or cubitus valgus. The spectrum of abnormalities is wide, as summarized in Table 1, which presents the prevalence of somatic manifestations. The absence of typical signs does not exclude the diagnosis, and subtle findings or isolated short stature may be the only presenting features.

Genetically, the most common karyotype is 45, X (40–50%), followed mosaic by forms such as 45, X/46, XX or 45, X/47, XXX (20–30%). Structural alterations include ring chromosomes, X isochromosomes, and deletions of Xp or Xq, frequently occurring in mosaic patterns. Despite clear cytogenetic classification, genotype–phenotype correlations remain insufficient, prompting investigation of epigenetic regulatory mechanisms influencing gene expression and contributing to clinical variability [20]. Notably, the retained X chromosome in 45, X individuals is of maternal origin in approximately 60–80% of cases [21].

Optimal medical care for individuals with TS requires multidisciplinary management throughout life, targeting endocrine, cardiovascular, skeletal, renal, audiological, and psychosocial domains. Growth hormone therapy is essential for promoting linear growth, while estrogen replacement—later combined with progestins—supports pubertal development and long-term health maintenance [22]. Systematic screening facilitates the early detection of comorbidities, with recommended assessments presented in Table 2 [7].

Given the chronic, multisystem nature of TS and substantial variation in clinical presentation, quality of life (QoL) has become an important measure complementing traditional clinical outcomes. QoL reflects not only physical health but also psychological, social, and functional dimensions, providing a comprehensive assessment of patient well-being. The aim of the present study was therefore to comprehensively evaluate and compare key determinants of quality of life in women with Turner syndrome and healthy controls. Using validated assessment tools, we sought to characterize physical, psychological, social, and functional domains to elucidate how TS affects overall well-being and daily functioning relative to the general female population.

## 2. Materials and Methods

### 2.1. Participation in the Study

This study included 30 women with a confirmed diagnosis of Turner syndrome and 43 age-matched healthy female controls. During the screening process, participants in the control group were confirmed to be free of chronic medical conditions. Inclusion criteria for the Turner syndrome group comprised: a clinical diagnosis confirmed by cytogenetic testing (karyotype analysis), age ≥ 18 years, ability to cooperate during questionnaire-based assessments, and provision of written informed consent. Exclusion criteria included lack of genetic confirmation of Turner syndrome despite phenotypic features, karyotypes other than monosomy X (45,X) or mosaicism 45,X/46,XX, significant musculoskeletal limitations preventing participation in assessments, age < 18 years, lack of informed consent, and cognitive or neurological disorders precluding cooperation.

The study was designed as an observational, comparative investigation and was conducted among patients attending the Gynecological Endocrinology Outpatient Clinic at University Clinical Hospital No. 1 of the Pomeranian Medical University in Szczecin between 1 December 2021, and 31 December 2024. All interviews and diagnostic procedures were performed by a single investigator to ensure methodological standardization and comparability of data.

The study protocol was approved by the Bioethics Committee of the Pomeranian Medical University in Szczecin (approval no. KB-0012/47/2021, dated 8 November 2021). All participants were adults and provided written informed consent prior to inclusion in the study.

### 2.2. Basic Procedures

Before the assessment, participants underwent a telephone interview and verification of their medical records. They subsequently completed a structured questionnaire including demographic data, medical history, results of genetic testing, details of hormonal therapy (growth hormone and estrogen–progestin therapy), menstrual history, the Beck Depression Inventory-II (BDI-II), and the standardized SF-36 questionnaire assessing health-related quality of life across eight domains (PF, RP, BP, GH, VT, SF, RE, MH).

In the initial analytical step, bivariate Spearman correlations were calculated between each SF-36 domain and six clinically relevant predictors: severity of depressive symptoms (BDI-II), number of comorbidities, hearing impairment, final height, perceived social support, and age at initiation of estrogen–progestin therapy. This exploratory approach allowed identification of SF-36 domains, showing statistically significant associations with at least one predictor and informed subsequent regression modelling.

The number of comorbidities was calculated as a simple count of chronic conditions documented in the medical history, including cardiovascular disease, renal anomalies, thyroid disorders, diabetes, osteoporosis, and hearing impairment. This pragmatic approach was used to reflect overall somatic burden rather than condition-specific effects; however, it treats heterogeneous conditions as equivalent and does not capture differences in disease severity or clinical impact.

Perceived social support was assessed using a single-item Likert-type question (rated 1–5) capturing the participant’s subjective perception of available emotional and practical support. This measure was intended as a brief indicator of perceived support rather than a comprehensive assessment using a validated multidimensional instrument.

Six clinical variables were subsequently entered into the regression analyses. Depressive symptom severity was measured using the BDI-II, with higher scores indicating more pronounced depressive symptoms. The number of comorbidities was established based on clinical history and medical documentation. Age at initiation of estrogen–progestin therapy was recorded in years, and final height was measured in centimeters during clinical examination. Hearing impairment was determined clinically and/or audiometrically and coded as 0 = absent or 1 = present. All predictors were treated as continuous variables except for hearing impairment, which was included as a dichotomous independent variable.

### 2.3. Statistical Analysis

Categorical variables were presented as counts and percentages, while continuous variables were expressed as means ± standard deviations (SD) and ranges. Between-group comparisons were conducted using the Mann–Whitney U test for continuous variables and the Chi-square test or Fisher’s exact test for categorical variables.

Multiple linear regression models were constructed for each of the eight SF-36 domains using a stepwise selection procedure, retaining predictors that improved model fit (preliminary threshold *p* < 0.10) or were clinically justified. Standardized beta coefficients (β) were reported to allow comparison of predictor effects across domains, and adjusted R^2^ values were calculated to assess overall model performance. Statistical significance was set at *p* < 0.05.

Given the relatively small sample size of women with Turner syndrome, all multivariable regression analyses were conducted with caution and were intended to be exploratory rather than confirmatory. Candidate predictors were selected a priori based on clinical relevance and previous literature rather than purely statistical criteria. The stepwise procedure was applied to limit model complexity and reduce the risk of overfitting; however, we acknowledge that this data-driven approach may yield unstable estimates. Accordingly, regression results should be interpreted cautiously and considered hypothesis-generating.

All multivariable regression analyses were performed exclusively within the Turner syndrome group. The control group was used solely for between-group comparisons of anthropometric, clinical, and quality-of-life measures and was not included in the regression models, as several predictors (e.g., comorbidity burden and hormone replacement therapy characteristics) are specific to Turner syndrome and not applicable to healthy controls.

## 3. Results

### 3.1. Characteristics of the Group

Among the 30 patients with Turner syndrome, 17 (56.7%) exhibited the classic monosomy X (45,X), and 13 (43.3%) showed mosaicism (45,X/46,XX). All 30 control participants presented a normal 46,XX karyotype.

#### 3.1.1. Anthropometric Parameters

Anthropometric characteristics, including age, height, body weight, and BMI, were compared between the TS and control groups (Table 3) using the Mann–Whitney U test. While age and body weight did not differ significantly, TS patients were significantly shorter than controls (mean height 152.5 ± 6.38 cm vs. 165.6 ± 5.46 cm, *p* < 0.00001). BMI values were slightly higher in TS patients, although the difference did not reach statistical significance.

#### 3.1.2. Education Level

Education level was compared between the Turner syndrome group and controls. Chi-square (χ^2^) tests were applied, and Fisher’s exact test was used when expected cell counts were low.

In terms of education, a higher proportion of TS participants had completed special or vocational schooling, whereas controls more frequently had secondary education. University-level education was more common among TS patients compared with controls. These differences were statistically significant (Table 4).

### 3.2. Hormonal Therapy and Menstrual History in Patients with Turner Syndrome

Most patients with Turner Syndrome received growth hormone (GH) therapy and estrogen–progestin (EP) treatment. The mean duration of GH therapy was 3.67 ± 3.24 years (range 0–10), and the mean age at the initiation of estrogen therapy was 14 ± 7.09 years (range 0–40). Menarche occurred at a mean age of 15.04 ± 2.30 years (range 11–19).

Among the 30 TS patients, 23 (76.7%) received GH therapy, 27 (90.0%) received EP therapy, and 26 (86.7%) were still on ongoing EP therapy at the time of data collection. Menstrual characteristics were assessed in 29 patients who had achieved menarche: 27 (93.1%) reported regular cycles, and 25 (86.2%) experienced medication-induced menstruation. These data are summarized in Table 5.

### 3.3. Comorbidities

Analysis of medical history revealed a significantly higher prevalence of hypothyroidism, cardiac defects, and hearing impairment in the TS group compared to controls. Hypertension and diabetes were more frequent in TS patients, but differences did not reach statistical significance. The detailed comparison of comorbidities between groups is shown in Table 6.

Comparison of the prevalence of comorbidities between TS subgroups (monosomy vs. mosaicism) did not reveal any statistically significant differences for the analyzed categories (*p* > 0.05).

### 3.4. Functional Status and Depressive Symptoms

The results of the SF-36 questionnaire in women with Turner syndrome (TS) and healthy controls are presented in Table 7. Compared with controls, TS patients demonstrated lower mean scores across most SF-36 domains, indicating poorer perceived functioning. Statistically significant between-group differences were observed for General Health (GH, *p* < 0.001), Vitality (VT, *p* = 0.002), Social Functioning (SF, *p* = 0.004), and Mental Health (MH, *p* = 0.01). Higher SF-36 scores indicate better health-related quality of life. All between-group comparisons were performed using the Mann–Whitney U test.

Differences in PF, RP, and RE domains did not reach conventional levels of statistical significance and should therefore be interpreted cautiously as non-significant trends rather than definitive group differences.

Beck Depression Inventory-II (BDI-II) scores are presented in Table 8. Women with Turner syndrome exhibited significantly higher levels of depressive symptoms compared with controls, with a mean BDI-II score of 10.2 ± 6.1 versus 5.1 ± 3.9 (*p* = 0.008). Although most TS patients fell within the range of minimal depressive symptoms, a notable proportion reported mild or moderate symptom severity.

### 3.5. Multivariate Regression Analysis of Quality-of-Life Domains (SF-36)

Multivariate regression analyses were conducted exclusively within the group of women with Turner syndrome (*n* = 30) to identify clinical, psychological, and social predictors of health-related quality of life as assessed by the SF-36 questionnaire.

Eight separate linear regression models were constructed, one for each SF-36 domain (PF, RP, BP, GH, VT, SF, RE, MH). Each model initially included six a priori selected, clinically relevant predictors: severity of depressive symptoms (BDI-II), number of comorbidities, hearing impairment, final height, perceived social support (single-item measure), and age at initiation of estrogen–progestin (EP) therapy. The number of comorbidities was treated as an indicator of cumulative somatic burden rather than the effect of individual medical conditions. Similarly, associations involving perceived social support should be interpreted in light of the brief, single-item nature of this measure. Table 9 presents only predictors retained in the final stepwise-selected models.

All models were constructed using a stepwise selection procedure applied to the predefined set of clinically relevant predictors. Standardized β coefficients describe the strength and direction of associations between predictors and SF-36 scores, with negative coefficients indicating poorer quality of life at higher predictor levels.

Across SF-36 domains, depressive symptom severity (BDI-II) emerged as the most consistent and strongest predictor, showing statistically significant negative associations in seven of the eight models (β range: −0.25 to −0.53). Its influence was particularly pronounced in psychological domains, including Role Emotional (RE) and Mental Health (MH).

In the physical domains (PF, RP, BP), the number of comorbidities was the dominant predictor, with standardized β values ranging from −0.30 to −0.40, indicating that greater multimorbidity was associated with poorer physical functioning. Hearing impairment was significantly associated with lower scores in General Health (GH) and Social Functioning (SF), suggesting a broader impact on perceived health status and social participation. Final height emerged as a significant positive predictor of Vitality (VT), indicating that taller stature was associated with higher perceived energy levels.

Perceived social support demonstrated consistent positive associations with several psychosocial domains, including Vitality (VT), Social Functioning (SF), Role Emotional (RE), and Mental Health (MH), highlighting its potential protective role for emotional and social well-being. Age at initiation of EP therapy contributed significantly to GH, VT, and MH, with later initiation associated with poorer quality-of-life outcomes.

Adjusted R^2^ values ranged from 0.28 to 0.42, indicating moderate but clinically meaningful explanatory power across models. The highest adjusted R^2^ was observed for the Mental Health domain (0.42), suggesting that psychological and social factors accounted for a substantial proportion of variance in mental health outcomes.

Given the limited sample size and the use of a stepwise selection procedure, the stability of the regression coefficients may be reduced. Therefore, these findings should be interpreted as indicative of potential associations rather than definitive or causal relationships and considered hypothesis-generating.

## 4. Discussion

The results of the present study are consistent with previous observations concerning quality of life and clinical characteristics in women with Turner syndrome (TS). In the studied cohort of 30 patients with TS, monosomy X was identified in 56.67% of cases, while mosaicism with a 45,X/46,XX karyotype was observed in 43.33% of patients. These proportions closely resemble those reported in the literature [1]. No other karyotypic variants were identified in the analyzed group.

Demographic and clinical data were obtained from medical records and a structured questionnaire completed by participants under the supervision of the study investigators. All women with TS had a genetically confirmed diagnosis and were under continuous endocrinological and multidisciplinary specialist care. The majority had received growth hormone and estrogen replacement therapy, with regular monitoring of hormonal status. This relatively homogeneous cohort with respect to diagnosis and standardized treatment provided a reliable basis for the assessment of factors influencing quality of life.

Comparative analyses of anthropometric parameters demonstrated no significant differences between women with TS and controls with respect to age, body weight, or BMI. As expected, height was significantly lower in the TS group (*p* = 0.00001), reflecting a hallmark feature of the TS phenotype related to SHOX gene haploinsufficiency, as previously reported by other authors [19]. Differences in educational attainment were also observed between groups. The TS group showed a higher proportion of individuals with special or vocational education and a lower proportion with secondary education, whereas secondary education predominated in the control group. The Chi-square test confirmed a statistically significant difference in educational level between groups (*p* = 0.00059). These differences should be considered as a potential confounding factor when interpreting between-group comparisons of quality of life, as educational attainment may influence health literacy, socioeconomic status, and access to resources. Similar conclusions can also be found in the work of Gould et al., where women with Turner syndrome currently achieve higher levels of education and employment than women in the United States as a whole [23].

TS is a rare condition characterized by hypergonadotropic hypogonadism, infertility, metabolic abnormalities, and an increased risk of autoimmune and cardiovascular diseases [24]. Advances in genetic research have demonstrated that phenotypic variability in TS cannot be explained solely by monosomy X, although SHOX remains the only gene convincingly linked to the core phenotype [25]. Cardiovascular abnormalities are a major clinical concern in TS and include aortic dilatation, bicuspid aortic valve, coarctation of the aorta, and hypertension [26,27]. TS is also associated with an increased risk of atherosclerotic cardiovascular disease and metabolic disturbances such as diabetes and dyslipidemia [28,29].

Growth hormone therapy is a standard component of childhood management in TS and has been shown to improve adult height, body composition, lipid profile, and cardiovascular parameters [30,31]. Hormone replacement therapy is equally essential for inducing puberty, supporting bone mineralization, and promoting the development of secondary sexual characteristics [32,33]. In the present study, women with TS initiated estrogen therapy later and experienced menarche at a significantly older age than controls (*p* < 0.00001), reflecting the delayed sexual maturation typical of TS due to primary gonadal insufficiency [34]. No significant differences were observed between karyotype subgroups with respect to duration of growth hormone therapy or timing of estrogen initiation and menarche, suggesting that treatment course may be influenced more by early diagnosis and standardized therapeutic protocols than by karyotype type alone.

Menstrual regularity did not differ significantly between the TS and control groups; however, the type of menstruation differed markedly. Most women with TS experienced pharmacologically induced cycles, while spontaneous menstruation predominated in controls. Spontaneous menstruation occurred primarily in women with mosaicism, in line with previous reports indicating partial preservation of ovarian function in this subgroup [35,36], although these differences did not reach statistical significance, likely due to limited sample size.

Consistent with existing literature, women with TS exhibited a significantly higher prevalence of hypothyroidism, congenital heart defects, and hearing loss compared with controls [26,37]. Hypothyroidism, most commonly autoimmune in origin, is reported in 30–70% of women with TS and requires lifelong monitoring [38]. The increased predisposition to autoimmunity has been linked to haploinsufficiency of immune-related genes located in the pseudoautosomal region of the X chromosome [39,40,41]. Cardiovascular anomalies, present in approximately 30% of the study cohort, represent a major determinant of morbidity and mortality in TS [42].

Hearing impairment, affecting both conductive and sensorineural pathways, is another frequent comorbidity in TS and may substantially reduce social functioning and overall quality of life [43]. The absence of significant differences between karyotype subgroups suggests that comorbidity risk is shaped more by hormonal, immunological, and environmental factors than by chromosomal constitution alone.

Women with TS are also at increased risk of depressive symptoms, influenced by hormonal dysregulation, medical burden, infertility, and psychosocial challenges related to self-esteem and social functioning. Depression has been linked to alterations in estrogen and testosterone levels and may worsen with delayed diagnosis or inadequate psychosocial support [44,45].

In the present study, women with TS reported significantly reduced quality of life compared with controls, particularly in the SF-36 domains of General Health, Vitality, Social Functioning, and Mental Health, along with higher levels of depressive symptoms [45,46]. These impairments were closely related to clinical factors, including comorbidity burden and hearing impairment. Despite widespread use of growth hormone and estrogen–progestin therapy, functional and psychological outcomes remained suboptimal, underscoring the long-term impact of chronic comorbidities.

These findings partially contrast with those, who reported near-normal quality of life in women with TS treated with growth hormone and induced puberty [46]. However, the present results align more closely with those of Amundson et al., who demonstrated persistent reductions in quality of life in adulthood despite earlier hormonal treatment, particularly in psychosocial domains [47]. Together with other reports, these findings emphasize that while somatic treatment is necessary, long-term multidisciplinary care addressing both medical and psychosocial dimensions is essential to optimize adult well-being in TS [48,49,50].

Several limitations of the present study should be acknowledged. The relatively small sample size reflects the rarity of TS but limits statistical power, particularly in multivariable analyses. Although clinically grounded predictor selection and stepwise regression were applied to reduce model complexity, this data-driven approach may yield unstable estimates and increase the risk of type I error. Therefore, the observed associations should be interpreted cautiously and regarded as hypothesis-generating. The comorbidity burden was operationalized as a simple count of chronic conditions, which does not capture disease severity or condition-specific effects. Similarly, social support was assessed using a single-item measure, which lacks the psychometric depth of validated multidimensional instruments. Finally, regression analyses were restricted to women with TS, enhancing disease-specific interpretability but limiting generalizability. Larger, preferably multicenter studies using comprehensive assessments are needed to confirm and extend these findings.

## 5. Conclusions

The analysis of the eight SF-36 domains in adult women with TS showed that quality-of-life impairment is multifactorial and encompasses both physical and psychosocial functioning, with the most affected areas being mental health, vitality, and social functioning, as detailed in Table 9. Multiple regression analysis further demonstrated that the severity of depressive symptoms (BDI-II) was the strongest predictor of reduced quality of life, underscoring the central role of psychological well-being in this population.

The findings also confirm the significant contribution of clinical factors to quality of life (Table 9). A higher comorbidity burden was associated with poorer general health, consistent with prior studies emphasizing multimorbidity as a key determinant of well-being in women with TS. The positive association between final height and vitality highlights the psychosocial relevance of external phenotype, as body image and stature are known to influence self-esteem and emotional functioning. Hearing impairment, which affects up to 20–40% of women with TS, was linked to decreased social functioning, reflecting the impact of communication difficulties on social engagement and interpersonal comfort. Additionally, poorer self-rated health was closely related to higher depressive symptom severity, reinforcing the importance of subjective health perception as a psychological indicator in this group.

Taken together, these results demonstrate that health-related quality of life in women with Turner syndrome is shaped by an interplay of somatic, psychological, and social factors. They highlight the need for a comprehensive, multidisciplinary care model that integrates cardiometabolic, endocrine, audiological, and mental-health support to more effectively address the complex needs of this population.

## Figures and Tables

**Table 1:** Somatic abnormalities reported in patients with Turner syndrome and their prevalence [1,19].

Somatic Abnormality	Gravholt (%)	Carvalho (%)
Short stature	95–100	83
Hypergonadotropic hypogonadism	>90	70
Delayed development in first year of life	50	–
Glucose intolerance	15–50	–
Type 2 diabetes	10	–
Thyroiditis/hypothyroidism	15–30	–
Hypertension	50	–
Elevated liver enzymes	50–80	–
Gluten intolerance	8	–
Inflammatory bowel disease	2–3	–
Otitis media	60	–
Hearing impairment	30	–
External ear deformity	15	44
Micrognathia	60	15
High-arched palate	35	46
Low posterior hairline	40	51
Broad, short-appearing neck	40	39
Pterygium colli (webbed neck)	40	25
Shield chest	30	18
Widely spaced nipples	5	40
Lymphedema of hands and feet	25	12
Multiple pigmented nevi	25	47
Nail hypoplasia/dystrophy	10	31
Vitiligo	5	–
Alopecia	5	–
Delayed bone age	85	–
Bone demineralization	50–80	–
Cubitus valgus	50	72
Short fourth metacarpal	35	71
Genu valgum	35	–
Congenital hip dislocation	20	–
Scoliosis	10	–
Madelung deformity	5	0
Bicuspid aortic valve	14–34	–
Aortic coarctation	7–14	–
Aortic aneurysm	3–42	–
Horseshoe kidney	10	0
Urinary tract anomalies ^1^	15	0
Renal aplasia	3	0
Emotional immaturity	~40	–
Learning difficulties ^2^	~40	–
Psychological and behavioral problems	~25	–

^1^ Abnormal position or duplication of renal pelvis, ureters, or vessels ^2^ Specific (nonverbal).

**Table 2:** Recommended screening and assessments for children and adults with Turner syndrome by age group [7,9].

Age (Years)	Recommended Assessments
0–4	Hip dislocation evaluation; Pediatric ophthalmology assessment (≥1 year)
4–10	Thyroid function tests (TSH, fT3, fT4, anti-TG, anti-TPO); Screening for celiac disease (TTG antibodies); Educational/psychosocial evaluation; Orthodontic assessment (≥7 years)
≥10	Thyroid function tests (TSH, fT3, fT4, anti-TG, anti-TPO); Screening for celiac disease (TTG antibodies); Educational and psychosocial evaluation; Orthodontic assessment; Ovarian function assessment/estrogen replacement therapy; Liver function tests, glucose, lipids, complete blood count, creatinine, blood urea nitrogen; Bone mineral density assessment (≥18 years)
All ages	Renal ultrasound; Hearing assessment by audiologist; Scoliosis/kyphosis evaluation; Education on TS and referral to support groups; Growth and pubertal development monitoring; Cardiovascular assessment by a specialist

TSH—thyroid-stimulating hormone; fT4—free thyroxine; fT3—free triiodothyronine; anti-TG—anti-thyroglobulin antibodies; anti-TPO—anti-thyroid peroxidase antibodies; TTG Ab—tissue transglutaminase antibodies; USG—ultrasonography; TS—Turner syndrome.

**Table 3:** Anthropometric parameters in the study and control groups and results of the Mann–Whitney U test.

Parameter	TS Group (*n* = 30)	Control Group (*n* = 43)	Test Statistic	*p*
Age (years)	Min–Max: 18–66 Mean ± SD: 33.67 ± 13.46 Median (IQR): 31.5 (21)	Min–Max: 22–65 Mean ± SD: 32.53 ± 11.87 Median (IQR): 26 (19)	U	0.83
Height (cm)	Min–Max: 137–165 Mean ± SD: 152.5 ± 6.38 Median (IQR): 153 (7)	Min–Max: 156–180 Mean ± SD: 165.6 ± 5.46 Median (IQR): 165 (8)	U	<0.00001
Body weight (kg)	Min–Max: 32–93 Mean ± SD: 59 ± 13.81 Median (IQR): 57.5 (20)	Min–Max: 45–97 Mean ± SD: 64.03 ± 11.28 Median (IQR): 62 (13)	U	0.10
BMI (kg/m^2^)	Min–Max: 15.58–39.73 Mean ± SD: 25.38 ± 5.81 Median (IQR): 24.97 (8.97)	Min–Max: 17.58–35.63 Mean ± SD: 23.32 ± 3.87 Median (IQR): 22.31 (4.49)	U	0.095

**Table 4:** Comparison of education level between TS patients and controls.

Education	TS Group (*n* = 30)	Control Group (*n* = 43)	*p* (χ^2^/Fisher)
Special school	2 (6.67%)	0 (0.00%)	0.00059
Vocational	3 (10.00%)	0 (0.00%)	—
Secondary	11 (36.67%)	35 (81.40%)	—
Higher education	14 (46.67%)	8 (18.60%)	—

**Table 5:** Hormonal therapy and menstrual characteristics in patients with Turner syndrome.

Parameter	TS Group (*n* = 30)	Mean ± SD (Range)/*n* (%)
GH therapy duration (years)	3.67 ± 3.24	0–10
Age at estrogen therapy initiation (years)	14 ± 7.09	0–40
Age at menarche (years)	15.04 ± 2.30	11–19
GH therapy received	23	76.7%
EP therapy received	27	90.0%
Ongoing EP therapy	26	86.7%
Regular menstruation *	27/29	93.1%
Medication-induced menstruation *	25/29	86.2%

* Menstrual data were available for 29 patients who had achieved menarche.

**Table 6:** Selected comorbidities in TS patients and controls.

Disease	TS Group (*n* = 30)	Control Group (*n* = 43)	*p* (Fisher)
Hypothyroidism	21 (70.0%)	8 (18.6%)	0.00002
Hypertension	8 (26.7%)	4 (9.3%)	0.061
Diabetes	2 (6.7%)	0 (0.0%)	0.17
Cardiac defects	9 (30.0%)	0 (0.0%)	0.00015
Hearing impairment	7 (23.3%)	0 (0.0%)	0.0013

**Table 7:** SF-36 questionnaire scores in TS patients and controls.

SF-36 Domain	TS (*n* = 30)—Mean ± SD	Median	Control (*n* = 43)—Mean ± SD	Median	*p*
Physical Functioning (PF)	68 ± 11	70	84 ± 9	85	0.07
Role Physical (RP)	72 ± 14	74	88 ± 10	89	0.06
Bodily Pain (BP)	76 ± 12	77	82 ± 11	82	0.12
General Health (GH)	58 ± 13	59	74 ± 12	75	<0.001
Vitality (VT)	54 ± 12	55	68 ± 11	69	0.002
Social Functioning (SF)	61 ± 11	60	79 ± 10	79	0.004
Role Emotional (RE)	73 ± 13	75	86 ± 12	87	0.09
Mental Health (MH)	66 ± 11	67	79 ± 10	80	0.01

**Table 8:** BDI-II scores in TS patients and controls.

BDI-II Category	TS Group (*n* = 30)	Control Group (*n* = 43)	*p*
Mean ± SD	10.2 ± 6.1	5.1 ± 3.9	0.008
Median (IQR)	9 (8)	5 (6)	—
Minimal (0–13)	69%	90%	—
Mild (14–19)	19%	10%	—
Moderate (20–28)	12%	0%	—
Severe (≥29)	0%	0%	—

**Table 9:** Multivariable linear regression models explaining SF-36 quality-of-life domains in women with Turner syndrome (*n* = 30).

Variable →/ Domains ↓	PF β (*p*)	RP β (*p*)	BP β (*p*)	GH β (*p*)	VT β (*p*)	SF β (*p*)	RE β (*p*)	MH β (*p*)
BDI-II	**−0.25 (0.048)**	**−0.28 (0.033)**	−0.20 (0.098)	**−0.36 (0.024)**	**−0.33 (0.036)**	**−0.28 (0.029)**	**−0.45 (0.005)**	**−0.53 (0.001)**
Social support	+0.12 (0.210)	+0.10 (0.280)	+0.14 (0.180)	+0.16 (0.090)	**+0.29 (0.022)**	**+0.34 (0.008)**	**+0.30 (0.015)**	**+0.31 (0.011)**
Number of comorbidities	**−0.35 (0.020)**	**−0.40 (0.011)**	**−0.30 (0.042)**	**−0.39 (0.015)**	−0.21 (0.229)	−0.17 (0.194)	−0.22 (0.128)	−0.18 (0.212)
Age at EP therapy initiation (years)	+0.05 (0.520)	+0.07 (0.410)	+0.09 (0.330)	+0.22 (0.041)	**+0.24 (0.035**)	+0.10 (0.260)	+0.12 (0.190)	+0.27 (0.028)
Final height (cm)	+0.18 (0.12)	+0.10 (0.42)	+0.06 (0.60)	+0.22 (0.110)	**+0.41 (0.014)**	—	+0.05 (0.67)	—
Hearing impairment (0/1)	−0.12 (0.34)	−0.15 (0.25)	−0.18 (0.17)	**−0.28 (0.049)**	—	**−0.44 (0.012)**	−0.18 (0.12)	−0.22 (0.153)
Adjusted R^2^	0.34	0.36	0.28	0.39	0.31	0.35	0.30	0.42

Statistically significant values (*p* < 0.05) are highlighted in bold. A dash (—) indicates that the predictor was not retained in the final model because it did not improve model fit. β values represent standardized regression coefficients, and adjusted R^2^ indicates the proportion of variance explained by each model after correction for the number of predictors. The horizontal arrow (→) next to “Variable” indicates the direction of the variables across the table. The vertical arrow (↓) next to “Domains” indicates the direction of the domains down the table.

## Data Availability

Data are available on special request after contacting author (M.K.).
